# Therapeutic use of Aldara™ in chronic myeloid leukemia

**DOI:** 10.1186/1479-5876-5-4

**Published:** 2007-01-25

**Authors:** Annette M Marleau, Jeffrey H Lipton, Neil H Riordan, Thomas E Ichim

**Affiliations:** 1 Medistem Laboratories Inc, Tempe Arizona, USA; 2 Department of Medical Oncology and Hematology, University of Toronto, Toronto, Canada

## Abstract

The potent clinical responses seen in patients with chronic myeloid leukemia (CML) after administration of donor-specific lymphocytes, as well as the correlation between the presence of antigen specific T cells and prolonged remission in these patients, suggests a role for the immunological control of CML. Here we propose Aldara™, a clinically used formulation of imiquimod, as an agent for augmenting immune responses to CML antigens. Our proposition is based upon 3 tenets: 1) Endogenous dendritic cells (DC) of CML patients, which are known to be derived from the malignant clone, express and present various leukemic antigens; 2) CML-antigen reactive T cell clones exist in the patient but in many situations are ineffectively stimulated to cause significant hematological responses; and 3) Antigen presentation by mature, activated DC, which endogenously express CML-antigens may endow the pre-existing ineffective T cell responses with ability to control CML progression. The practical use of Aldara™ as a localized activator of DC in the context of present day leukemic therapeutics, as well as various properties of this unique immune modulator will be discussed.

## Background

Chronic myeloid leukemia (CML) is a myeloproliferative disease characterized by an indolent chronic phase during which immature myeloid cells increase in the peripheral blood and bone marrow, followed by an accelerated phase, associated with resistance to standard therapies, and terminates in blast crisis where undifferentiated blasts damage vital organs, leading in death. Treatment of CML has undergone several major advancements: a) The development of chemotherapeutic interventions such as busulphan and 6-thioguanine in 1953; b) The introduction of alpha interferon in 1983; c) Bone marrow transplantation in 1986; and d) BCR-ABL-specific tyrosine kinase inhibitors in 1998 [1]. Therapeutic interventions for CML aim to accomplish three goals: to achieve a hematologic remission (normalization of leukocyte numbers), to achieve cytogenetic remission (0% Ph-positive cells on chromosomal analysis), and, to achieve molecular remission (negative PCR result for the BCR-ABL fusion transcript) [2]. The current standard of care for CML patients is administration of imatinib, a selective inhibitor of BCR-ABL, or allogeneic stem cell transplantation [3]. Although imatinib induces hematological and sometimes even cytogenetic remission in the accelerated phase [4] and in myeloid blast crisis [5], these remissions are often short-lived, due in part to the ability of the CML cells to mutate. In a study where imatinib-treated patients were followed for 4.5 years, it was reported that hematologic resistance occurred in 25%, 41%, and 92% of patients in CP, AP, and myeloid BC, respectively, and was associated with BCR-ABL mutations in 45% of patients [6]. Generally, resistance to imatinib is associated with mutations in the ATP-binding pocket of the BCR-ABL kinase, and also with several other factors: 1) Amplification of the BCR-ABL transcript [7]; 2) Expression of drug efflux proteins such as P-glycoprotein [8]; and 3) Increased plasma concentrations of the imatinib binding protein, alpha -1 acid glycoprotein [9]. In light of these limitations, as well as the fact that only a small subset of patients are eligible for bone marrow transplantation, strong incentive exists for development of novel approaches to CML therapy.

## Immunogenicity of CML: The Adaptive Immune Response

The concept that leukemic cells are immunogenic was introduced in the 1960s when Mathe's group demonstrated a survival advantage in acute lymphocytic leukemia (ALL) patients that were treated with irradiated allogeneic blast cells together with BCG and chemotherapy, in comparison to patients receiving chemotherapy alone [10]. Similarly, in a 1975 study of 50 acute myelocytic leukemia (AML) patients induced into remission, those receiving irradiated allogeneic blasts together with BCG in combination with chemotherapy had an average survival of 510 days compared to patients receiving chemotherapy alone who had an average survival of 270 days [11]. Despite these positive results, immunotherapy fell out of favor when a meta-analysis of 24 trials concluded no clinically relevant benefit in 1983 [12]. Immunogenicity of CML cells was supported by reports of antibody [13] and T cell proliferative [14] responses in CML patients after administration of irradiated allogeneic cells together with immunological adjuvants. Furthermore, administration of purified IgG antibodies from goats immunized with the human CML cell line K562 in two CML patients led to a sharp decrease and the eventual eradication of blasts from the peripheral blood and bone marrow [15]. Although this therapeutic option cannot be advocated due to the potential for induction of serum sickness, it does suggest the existence of CML-specific antigens.

Molecular evidence for the existence of CML-specific T cell responses was brought forward by Cheever et al who demonstrated that CD4 T cell immunity could be generated toward BCR-ABL expressing cell lines after vaccination of mice with peptides generated from the junctional region of the BCR-ABL fusion protein [16]. Indeed, the identification of HLA class I [17], and HLA class II- binding [18] BCR-ABL peptides supports the hypothesis that antigen presentation, and subsequent T cell recognition of this specific oncoprotein may serve as a mechanism for the graft versus leukemia (GVL) effect commonly seen in bone marrow transplant patients. These studies also demonstrated that presentation of BCR-ABL peptides is mediated only by specific HLA haplotypes. Since patients lacking such haplotypes also can mount a GVL response, it is clear that BCR-ABL peptides are not the only antigens involved. However, there exists an epidemiological correlation between patients having the same HLA-haplotypes that are known to bind the BCR-ABL peptides, such as HLA-A3, HLA-B8, and HLA-DR4, and resistance to developing CML [19-21]. The identity of other peptides that are presented by these alleles in the context of CML is still under investigation.

Utilization of synthetic peptides corresponding to the BCR-ABL junctional areas as immunogens has been performed by numerous groups to activate both CD4 and CD8 T cells from healthy donors [17,18,22]. Interestingly, in vitro generated CD4+ T cells specific to BCR-ABL immunogenic peptides were shown to proliferate and produce Th1 cytokines in response to HLA-matched CML blasts in absence of exogenous antigen presenting cells [23]. This finding complements another report that CML progenitor cells are CD34+ MHC II+ and can directly present antigens to T cells [24]. A fundamental question is whether T cells from CML patients under natural conditions can recognize BCR-ABL antigens, or whether through a mechanism of tumor-induced tolerance, these cells are anergized or deleted, as occurs in melanoma [25]. Indeed, T cell anergy has been reported in a series of experiments involving the stimulation of lymphocytes with autologous CML cells [26]. Functional escape from unresponsiveness was demonstrated by incubation of the cultures with IL-2. When precursor frequencies were evaluated, it was determined that a much lower number of CML-specific CD8 T cells (1:38,000) was observed in comparison to CD4 cells (1:4,000). Whether this is a result of preferential induction of apoptosis in CD8 cells or whether CD4 cells are capable of inducing effective CML-inhibitory responses is not known. However, in the context of bone marrow transplantation (BMT), it is known that CD8-depleted donor lymphocyte infusion is as effective as non-depleted lymphocytes at inducing remission in post BMT relapse [27], therefore it appears that at least in some systems, CD4+ cells specific for leukemic antigens may be therapeutically beneficial.

While the concept of generating CML-specific T cells is appealing, the question of why such T cells don't naturally control CML arises. As previously mentioned, it appears that there is a functional incapacitation of leukemic-specific T cells in patients. Reports of T cell anergy in CML patients to specific leukemia-associated antigens have included the demonstration of an inability to raise BCR-ABL-targeting T cells from PBMCs derived from CML but not healthy volunteers [28], as well as the reduction in number of T cells specific for the CML antigen PR1 in interferon non-responding patients [29]. These observations raise the question if the T cells exist in a state of anergy from which they can be rescued by immune stimulation, or whether they are ablated by central tolerance or peripheral apoptosis. Evidence seems to point to the former rather than the latter. Specifically, administration of IFN-alpha to patients has been demonstrated to induce/restore ability of BCR-ABL peptide specific T cells to produce Th1 cytokines, as well as to endow them with cytotoxic activity [30]. When looking at the second well-known CML-specific antigen PR-1, it was demonstrated that patients entering cytogenetic remission through administration of IFN-alpha also had a re-emergence of high-affinity antigen-specific CD8 cells with potent cytolytic activity in vitro [31].

From these studies it appears that not only is T cell immunity possible to CML cells, but such immunity appears to be relevant to clinical outcome. The fact that antigen-specific suppression of anti-CML responses occurs [28,31] but can be reversed by appropriate immune stimulation provides impetus for studies investigating immune modulatory therapies for CML.

## Immunogenicity of CML: The Innate Immune Response

In recent years the molecular identification of mechanisms associated with innate recognition of "danger" signals has led to a rebirth of the study of innate immunity. Specifically, the discovery of the toll-like receptors (TLRs) as well as other "sensors of danger" such as the nucleotide-binding oligomerization domain (NOD)-containing proteins has stimulated a plethora of studies assessing the role of innate immune components in areas not traditionally associated with immunology. For example, TLRs have been shown to play a critical role in situations ranging from ischemia-reperfusion injury [32], to heart failure [33], and tumor regression [34]. In terms of CML, it is known that various innate immune cells such as macrophages [35] and NK cells are capable of spontaneously lysing leukemic cells. As a matter of fact, the classically used cytotoxicity assay for determination of NK activity involves using the CML blast crisis-derived cell line K562 as a target [36].

NK cells lyse targets that possess low or absent levels of MHC class I molecules [37]. This concept was termed the "missing self" hypothesis and was molecularly demonstrated by the fact that NK cells possess inhibitory receptors that transduce a negative signal upon ligation of MHC alleles [38]. Therefore, the NK cell is thought to act as a "back-up" mechanism of immune surveillance against neoplasia: Specifically, tumors which lose MHC expression in order to evade T cell mediated immune attack can not escape since the loss of MHC will sensitize them to killing by NK cells. The problem with this hypothesis was that certain cell types such as erythrocytes do not express MHC but are not target of NK lysis. To deal with this, activatory receptors were subsequently discovered on NK cells such as NKG2D whose ligand is MICA, a distant homolog of major histocompatibility complex (MHC) class I whose expression is associated with neoplasia, infection or sub-lethal cellular damage [39]. The important role of NKG2D in immune surveillance was demonstrated in studies where the neutralization of NKG2D resulted in enhanced incidence of spontaneous tumor formation in a murine model [40]. MICA expression is associated not only with cellular stress but also with DNA damage and activation of DNA-repair proteins such as ATR, ATM or Chk1 [41], proteins which are chronically activated during the process of carcinogenesis [42]. In respect to CML, CD34+ cells from patients but not from healthy volunteers were demonstrated to expressive high levels of MICA and MICB, which was associated with activation of NKG2D and lysis by NK cells [43]. Supporting the role of MICA in CML are studies that demonstrated that transfection of various cells with BCR-ABL actually increases the concentration of MICA [44]. This upregulation of MICA correlated with ability of the transfected cells to activate NK cells to proliferate, produce IFN-γ, as well as lyse BCR-ABL expressing cells.

The role of NK cells in CML is very interesting. One reason is that NK cells are found naturally residing in the bone marrow and affect hematopoiesis through secretion of stimulatory factors such as GM-CSF [45], as well as their ability to produce TNF-α and IFN-γ [46], which inhibit hematopoiesis. The functional activity of NK cells to alter hematopoiesis was demonstrated in an experiment where the transfer of activated NK cells with bone marrow progenitors into lethally irradiated syngeneic mice resulted in greater engraftment in the recipients in comparison to mice receiving bone marrow and control cells [47]. In light of the fact that IL-2 activated NK cells have been demonstrated to specifically lyse not only CML cell lines but also primary blasts [48], and the observation that activated NK cells home into the bone marrow [49], the therapeutic possibilities of this cell population have attracted much investigation in CML.

Ex vivo activation of NK cells by IL-2 was performed in bone marrow cultures as a method of "purging" AML cells from the non-malignant marrow for use in autologous bone marrow transplant resulting in clinical remission in a subset of patient [50]. Additionally, the NK cell line NK-92 was generated under good manufacturing practices with the purpose of ex vivo purging of CML cells for autografts due to its selective ability to lyse leukemic progenitors [51]. In terms of in vivo administration of activated NK cells, to our knowledge no studies have been conducted in CML patients, however use of NK stimulating agents such as IL-2 [52,53], and roquinimex [54], has been performed with some clinical benefit, although at present these therapies have not been optimized. Despite this, the clinical relevance of NK cells in CML should not be underestimated. Early studies have demonstrated that peripheral blood CD56+, CD16+ NK cells derived from CML patients were inferior to cells purified of a similar phenotype from healthy controls in terms of cytotoxic activity towards K-562 cells, as well as IFN-γ and IL-1 production in response to PHA [55]. More recent studies have shown that CML patients who respond to interferon [56] or other immune modulatory therapies such as heat shock protein vaccination generally have higher CD56+, CD16+ NK cell mediated cytotoxic activity towards K562 cells as compared to treatment non-responders [57]. An interesting observation is that as a last-ditch effort by the immune system to preserve the host, NK cell activity in blast crisis phase of CML is reported to be increased in comparison to healthy controls [58]. A practical explanation may be that the high concentrations of circulating NK activatory compounds may be systemically activating the NK cells.

In conclusion, while the effector innate immune system definitely is involved in the control of CML, little work has been performed in this area. The essential role of cells like DC and NK in terms of shaping the T cell response is only now beginning to be elucidated. Future studies integrating the activation of both the innate and adaptive immune responses will lead to development of novel therapeutics. What follows is a hypothesis of a novel way of harnessing the innate and adaptive immune responses through the use of a clinically available agent, Aldara™.

## Aldara™ as an Immune Stimulant

The active ingredient in Aldara™ is imiquimod, a low molecular weight immune stimulant. Previous to development of Aldara™ as a topical form of imiquimod, oral imiquimod has been assessed clinically for immune stimulatory effects both in cancer and HIV patients in the 1990s. These studies have demonstrated an unacceptable benefit/toxicity profile and as a result administration via the topical route was chosen for development. Specifically, Goldstein et al reported a Phase I study involved 12 patients with HIV infection that were dose escalated by 100 mg/week until toxicity was reached. When toxicity was reached, maintenance dosing of 100 mg/week lower than the toxic dose was performed. Dose-limiting toxicity occurred in 3 patients at 200-mg, 5 at 300-mg, and 3 at 400-mg dose levels. One patient tolerated the 500-mg dose without dose-limiting toxicity. While 7 of the 12 patients completed the 12 week dosing schedule, no consistent effect on viral load was observed. Importantly, serum interferon alpha was observed to rise, as well as neopterin and beta2-microglobulin, which are markers of immune activation [59]. In cancer therapy, Witt et al observed no clinical responses in 14 patients receiving 100–500 mg of imiquimod on a once or twice weekly basis. In agreement with the HIV study, dose limiting toxicities were observed between 300–500 mg week, and serum markers of immune activation were elevated. The authors concluded that before Phase II trials begin, establishment of a consistent tolerated dose and administration schedule is needed [60]. Savage et al used daily oral treatment of 25–200 mg of imiquimod in patients with refractory cancers. Of 21 patients in the study, one patient had a partial clinical response in disease stabilization. Only three of the 21 patient completed the scheduled treatment course of 112 days. These patients received the 50 mg/day dose, all other patients dropped out due to toxicity, death, or personal reasons. The authors concluded that although elevation in plasma markers of immune activation were observed, the treatment schedule used needed to be modified due to high levels of toxicity [61]. Given the impracticality of using systemic oral imiquimod therapy, the topical form of imiquimod was developed for dermatological uses under the name Aldara™.

Aldara™ is a formulation of 5% imiquimod in an oil-in water vanishing cream base that is approved for use in the United States for treatment of actinic keratosis, superficial basal cell carcinoma and external genital warts [62]. Aldara™ is supplied in single-use packets of 250 mg of cream, of which are applied 2–5 times per week depending on indication. Specifically, for treatment of actinic keratosis (AK), a premalignant dysplasia of the epidermis associated with UV exposure, Aldara™ is applied on a twice-weekly basis to a 25-cm2 treatment area surrounding the lesion on the face or scalp for no more than 16 weeks. In two pivotal studies submitted for FDA-registration, of a total of 215 actinic keratosis patients treated as described, 97 patients had complete resolution of lesions. This is in contrast to the 221 patients receiving placebo cream in which only 7 patients reported clearance of lesions [62]. Of the 185 patients with superficial basal cell carcinoma treated with Aldara™ in the FDA pivotal trial complete responses were observed in 139 patients, whereas of 179 patients treated with control only three patients responded. The treatment scheme for this study, which is in clinical use today, involves topical administration of 250 mg of cream for 5 days a week for the period of 6 weeks (62). The third accepted indication, external genital warts, was approved based on a trial of 109 treated patients, of which 54 had complete clearance, whereas of the 100 patients treated with vehicle control only 11 had clearance. The accepted treatment schedule involves 3 times per week topical treatment for a maximum of 16 weeks [62].

In addition to the above date, subsequent data supporting effectiveness of Aldara™ for the above indications were reported. For example, Lee et al examined 146 AK patients which were administered Aldara™ either 2 or 3 times per week for a period of 16 weeks. Recurrence of AK lesions was observed in only 24.7% (19 of 77) of the patients on the 3 times per week regimen and in 42.6% (23 of 54) of the patients on the 2 times per week regimen. With exception to localized irritation, no adverse effects were reported in any of the patients in this 30 center study [63]. The importance of immune modulation was also observed in patients receiving Aldara™ for this indication in that another study demonstrated lesional infiltration of CD11c+ dendritic cells, as well as both CD4 and CD8 T cells in AK patients responding to Aldara™ on the 3 times per week regimen [64]. In a study evaluating long-term responses of superficial basal cell carcinoma patients to Aldara™ treatment, 169 patients were treated using the currently accepted 5 days a week, for the period of 6 weeks, schedule. On average, the clearance rate at 12 weeks post completion of treatment was 94.1%, and at the 2-year follow-up was 82.0% [65]. Results in the treatment of genital warts have also reproducing the initial findings in the pivotal studies. For example, Arican et al reported a double blind study treating 34 patients on the accepted 3 times a day for 12 weeks regimen. Of these, 23 patients achieved complete clearance of the treated warts, whereas only 1 out of the 11 patients who received placebo cream had wart clearance [66]. Collectively, these studies attest to the reproducibility of the clinical relevance of this topical immunomodulatory agent in a variety of dermatological settings.

Although the first FDA approval for use of Aldara™ occurred in 1997, the molecular mechanism of action was only identified five years later in 2002 by Hemmi et al, who demonstrated that active ingredient, imiquimod, is a stimulator of the toll-like receptor 7 (TLR7)-MyD88-dependent signaling pathway on immune cells [67]. Other stimulators of TLR-7 are known such as isatoribine [68] and resiquimod [69] although a discussion of these compounds is outside of the scope of this review. Original analysis of TLR-7 expression revealed pulmonary, placental and splenic tissues as high expressers [70]. At a cellular level it was observed that the primary interferon alpha producing cell, the plasmacytoid DC, responds to treatment with imiquimod by upregulating the lymphoid homing receptor CCR-7, enhancing expression of costimulatory molecules, as well as producing large amounts of interferon alpha [71]. In the mouse, plasmacytoid DC found in the skin are positive for CD4, GR-1, B220, and MHC class II. These cells are found in the skin of healthy mice, and dermal interferon response to imiquimod correlates with amount of these cells in the skin [72]. In humans treated with Aldara™, regardless of whether treatment is for superficial basal cell carcinoma, Bowden's disease, or cutaneous T cell lymphoma, all patients develop an increase in local plasmacytoid DC having the phenotype CD123+ and BDCA-2+ and associated with a specific cytokine signature [73]. The authors of the study reported that these cells are found in small quantities in healthy volunteers, supporting previous studies also describing plasmacytoid DC at the border between the basement membrane and dermis [74].

The importance of these DC in priming of immune responses subsequent to imiquimod administration was demonstrated in experiments applying a combination of imiquimod and antigen epicutaneously. This type of "trans-immunization" was able to elicit potent cytotoxic T cell responses through the activation of both plasmacytoid, and myeloid-derived DC in a murine model [75]. Although some in the field believe that the murine system possesses certain peculiarities that may not allow direct comparison with results in human studies, an interplay between the plasmacytoid DC and the activation of immature myeloid DC was demonstrated in individuals administered Aldara™ cream. The immature myeloid DC administered to Aldara™ -treated subjects migrated faster and more efficiently to draining lymph nodes than mature myeloid DC and control treated immature myeloid DC [76].

In terms of its clinical use, an immunological mechanism is believed responsible, especially in cases of virally-induced genital warts since imiquimod itself is not active against viruses in the absence of a functional immune system [77]. Indeed, patients with genital warts, which exhibit high responsiveness to Aldara™, often display a higher level of plasmacytoid DC activation, as judged by expression of HLA-DR than non-responders [77]. On the T cell side, an increased number and level of IFN-γ production is associated with response in patients with intraepithelial neoplasia [78]. Overall, the antiviral and anti-neoplastic/preneoplastic activities of imiquimod are believe to work according to the following steps: a) Initial activation of low level pro-inflammatory cytokine secretion by resident macrophages and local plasmacytoid DC; b) Chemoattraction of additional plasmacytoid DC from circulation; c) Migration of Langerhans cells to draining lymph nodes/NK production of IL-12, and d) Increased presentation of antigen from DC, resulting in T cell activation [79].

In summary, Aldara™ has been demonstrated to induce various molecular and cellular changes that evoke clinical responses against various viral and preneoplastic/neoplastic conditions. For the remainder of the manuscript we will discuss the therapeutic possibility of medication in regards to CML.

## Aldara™ Stimulation of CML-Specific Responses

CML is a unique disease from the perspective of the tumor immunologist. Since the leukemic clone differentiates into all cells of the myeloid lineage, the majority of DC isolated from CML patients direct progeny of the leukemic stem cell. It is established that in CML patients, DC can not only present the leukemia specific BCR-ABL peptides, but also that such presentation can lead to generation of antigen-specific T-cells capable of inducing lysis of CD34 cells from CML but not control patients in an MHC-I dependent manner. Indeed, CML is different than almost any other cancer in that DC immunotherapy would not require the pulsing of DC with exogenous antigens, since CML DC endogenously express BCR-ABL immunogenic peptides at sufficient concentrations to stimulate a T cell response [80]. This concept has been demonstrated clinically through the use of GM-CSF administration to expand endogenous DC numbers followed by donor lymphocyte infusion, resulting in a positive impact on patient survival that was associated with increased anti-leukemic response [81], which was believed to be due to enhanced presentation of leukemic antigens from DC. However the treatment proposed here is less "invasive" and more applicable to widespread implementation.

The proposition of this paper is that topical administration of Aldara™ would induce the differentiation and expansion of CML DC at the cutaneous sites of administration. This differentiation would then lead to upregulated expression of MHC I and II bearing leukemic peptides, costimulatory molecules, as well as interferon alpha production, in a milieu that would not only rescue the CML-specific T cells from anergy, but also induce their proliferation, expansion, and acquisition of cytotoxic/leukemia inhibitory function. Several questions arise...

First, will the DC that are activated by Aldara™ treatment actually express BCR-ABL peptides? Some believe that plasmacytoid DC are only derived from lymphoid lineage and thus will be BCR-ABL negative. To this question we respond by stating the main anti-CML effect of the Aldara™ treatment will not be through the direct activation of the plasmacytoid DC, but through the indirect activation of the myeloid derived DC that occurs as a result of the immune stimulation. For example it was demonstrated that not only does Aldara™ treatment potently induce maturation of myeloid DC through indirect effects [82], but also that local treatment induces migration of such cells to spleen, where in conjunction with NKT cell activation results in induction of systemic immune modulation capable of promoting antigen-specific immunity in a melanoma model [83]. Therefore we anticipate that the activation of myeloid DC will induce an increased presentation of anti-leukemic T effector cells. Additionally, the known effects of Aldara™ in activating NK cell function are also postulated to contribute to anti-leukemic effect [84].

Second, will the CML-specific T cells not be anergized in vivo, such that even if the presentation of BCR-ABL peptides does occur, the T cells will not be able to mount a productive anti-leukemic response? Although it was previously stated that anergy to CML-specific T cells occurs in CML patients, this anergy seems reversible either by addition of cytokines or antigen-specific vaccination. The recent reports of hematologic and cytogenetic remissions induced by immunization with BCR-ABL peptides support this notion. Interestingly it was observed that higher antigen-specific responses occur in patients that are concurrently receiving interferon alpha [85-87]. These observations suggest that T cell clones specific for CML may indeed by reactivated in vivo, in a similar manner to how ex vivo IL-2 treatment of T cells induces escape from anergy [88]. Indeed imiquimod has been demonstrated to increase expression of CD80 and CD86, which are involved in escape from anergy [89,90], as well as upregulate production of IL-12 [91] which inhibits IL-10 generation [92], known to be responsible for maintaining the anergic state [93]. Along these lines, it is reported that upregulation of costimulatory molecules on circulating tumor cells in CLL patients following topical administration of imiquimod occurs, thus supporting the possibility that local administration of this agent may have systemic effects [94].

Third, what about CML specific antigens that are not expressed endogenously in DC of CML patients? Specifically, antigens such as telomerase [95], PR1 [29], PASD1 [96], CML28 and 66 [97] have not been identified in CML derived DC although immune responses to these have been observed in patients. To this we first answer that healthy, non-leukemic derived DC should theoretically engulf and present antigens derived from leukemic cells due to their "immunological sentinel" role. Therefore the activation of DC will allow, to some extent, presentation of leukemic antigens that are non-endogenous to the DC. Secondly, we argue that although numerous CML antigens exist that would not be present in leukemia-derived DC, BCR-ABL, which is present, is known to be clinically relevant based on responses observed in patients immunized with BCR-ABL-derived peptides [17,87]. Thirdly, we believe that the general immunostimulatory activity of imiquimod will assist the immune response in "de-anergizing" itself towards relevant antigens. For example it is known that CML patients have lower NK cell activity in comparison to healthy controls [55], and that clinical response to immunotherapy is associated with enhanced NK activity [56,57], therefore the NK stimulatory activity of Aldara™ therapy may in fact actually mediate enhanced anti-CML NK activity. An interesting but undeveloped area would be the effect of imiquimod on the immune suppressive CD4+ CD25+ T regulatory cell population. These cells mediate inhibition of T cell activation and effector function both locally and systemically [98], and are responsible for the blunted immune response to numerous malignancies [99-102]. Antileukemic immune therapies are enhanced by depletion of this cell population [103,104]. Recently it was demonstrated that TLR agonists function to render T cells unresponsive to the suppressive effects of T regulatory cells [105], as well as actually inhibiting the ability of T regulatory cells to suppress immune responses, and anti-tumor immunity [106]. Although it is unlikely that plasma levels of imiquimod are able to systemically derepress immunity by inhibiting CD4+ CD25+ T cell functions, the effects at the local site of administration may be interesting to examine.

## The Clinical Implementation

How will Aldara™ administration be optimally used clinically? In the ideal situation, Aldara™ would be administered as maintenance therapy after induction of molecular remission using bone marrow transplant, IFN-alpha or imatinib. The low cost and tolerability of Aldara™ would make this an interesting immunostimulating adjuvant. Another situation where this therapy may be useful is patients whom are non-responsive to imatinib and cannot tolerate the effects of INF-alpha. Yet another embodiment of this therapy would be application to the skin area where CML-specific vaccines are administered, however, it has been demonstrated that the vaccine stimulating effects do not necessarily require local administration [83], although this is controversial since it has only been demonstrated in mice. Dosage could begin based on the standard protocol for superficial basal cell carcinoma of 5 times per week on a 25 cm^2 ^area of skin. Monitoring of therapy would most likely involve ex vivo analysis of cytokine production, as well as in more advanced clinical research settings tetramer analysis for BCR-ABL specific T cells.

A potential concern for clinical implementation of Aldara™ treatment for CML may be the possibility of activation of immunity that targets not only hematopoietic stem cells of leukemic origin, but also non-leukemic hematopoietic stem cells. Indeed the graft versus leukemia effect observed after donor lymphocyte infusions is correlated with a period of aplasia in which normal hematopoietic stem cells are also targeted [107]. This is in agreement with other studies of immune stimulation to cancer antigens in which autoimmunity arises as an unintended adverse event [108]. Given that Aldara™ treatment as used clinically today is not associated with induction autoimmunity towards hematological targets, the authors do not anticipate this to be a major concern, however the experimental use of Aldara™ in CML patients will need to be monitored by hematological assessment to ensure that CML-directed responses do not crossreact with non-malignant progenitor cells.

## Conclusion

We present a novel hypothesis that administration of the topical TLR-7 agonist imiquimod in the form of Aldara™ may be a useful immunotherapy/adjuvant therapy for CML patients with minimal residual disease. Thus a scientific basis for utilization of this generally innocuous medication is proposed for a lethal disease. Although the current concept may require various modifications before clinical entry, we put forth this idea as a stepping stone for other investigators to expand upon. The lack of significant toxicity and widespread use of this medication makes this hypothesis attractive for testing. Present day therapies for imatinib-resistant, IFN-alpha non-eligible, CML patients are limited to maintenance therapy on hydroxyurea or busulfan. The administration of Aldara™ to this patient subgroup may provide a novel means of life extension without drawbacks of severe myeloablative protocols.
